# Volatile organic compounds in exhaled human breath for the diagnosis of malignant pleural mesothelioma: a meta-analysis

**DOI:** 10.3389/fonc.2025.1537767

**Published:** 2025-05-28

**Authors:** Tingting Zhao, Xiao Hu, Minghua Peng, Wei Wei, Ninghua Fu, Chen Chen, Zhenguang Chen

**Affiliations:** ^1^ Department of Thoracic Surgery, Guizhou Hospital of the First Affiliated Hospital of Sun Yat-sen University, Guiyang, Guizhou, China; ^2^ Department of Oncology, the School of Clinical Medicine, Guizhou Medical University, Guiyang, Guizhou, China; ^3^ ChromX Health Co. Ltd., Guangzhou, Guangdong, China; ^4^ Department of Thoracic Surgery, the First Affiliated Hospital of Sun Yat-Sen University, Guangzhou, Guangdong, China

**Keywords:** volatile organic compounds, malignant pleural mesothelioma, exhaled breath, diagnosis, meta-analysis

## Abstract

**Objective:**

Malignant pleural mesothelioma (MPM) is a relatively rare thoracic tumor with a high mortality rate, making early diagnosis and treatment challenging. The present study evaluated the utility of exhaled volatile organic compounds (VOCs) in diagnosing MPM.

**Methods:**

The Cochrane Library, PubMed, EMBASE, and Web of Science were systematically searched for clinical trials assessing the diagnostic ability of VOCs for MPM through August 30, 2024. Quality was evaluated using the QUADAS-2 tool. A meta-analysis was performed with a bivariate model for sensitivity and specificity using Stata MP 17.0 software.

**Results:**

Eight trials with 859 subjects were included. VOCs were found to have a pooled sensitivity of 0.86 (95% confidence interval [CI] 0.75–0.93), a pooled specificity of 0.73 (95% CI 0.58–0.84), and an area under the curve (AUC) of 0.88 (95% CI 0.85–0.90) in differentiating MPM patients from healthy controls. In addition VOCs had a pooled sensitivity of 0.89 (95% CI 0.83–0.93), a pooled specificity of 0.79 (95% CI 0.57–0.91), and an AUC of 0.91 (95% CI 0.88–0.93) in differentiating MPM patients from asymptomatic individuals formerly exposed to asbestos (AEx).

**Conclusions:**

Although the utility of VOCs in diagnosing MPM varied among clinical trials, VOCs in exhaled human breath may have a potential role in the diagnosis of MPM. Large-scale randomized clinical trials are warranted.

## Introduction

Malignant pleural mesothelioma (MPM) is an aggressive and frequently fatal type of thoracic tumor closely associated with asbestos exposure (AEx). The GLOBOCAN database estimated that 30,618 patients were newly diagnosed with MPM in 2022, with high mortality rates (1). MPM is likely to arise in developing countries where asbestos was manufactured and utilized in industrial development (2). Moreover, despite asbestos being banned in western European countries during the second half of the 20^th^ century, MPM rates remain high due to the long latency period (40–50 years) between initial AEx and MPM diagnosis (2, 3). MPM has also been linked to exposure to other environmental agents, but this has been less extensively studied (4).

Due to its relative infrequency, lack of comprehensive research, and relative inexperience of clinicians, MPM diagnosis and management remain difficult. The median survival for patients with MPM has been reported to range from 3–12 months (5). This grim prognosis has been primarily attributed to late diagnosis at advanced stages and high misdiagnosis rates due to the absence of specific symptoms and reliable biomarkers. Despite promising advances in the diagnosis and treatment of MPM, the available options remain limited (6), indicating the critical need for earlier detection and intervention. Although enhanced computed tomography (CT) of the chest has been the preferred imaging diagnostic modality for MPM, low-dose spiral CT screening was not effective in lowering the mortality rate of MPM in high-risk individuals and lacks sufficient specificity and sensitivity (7–10). A definitive diagnosis of MPM requires invasive procedures, including pathological and/or cytological examinations (11). Despite promising initial results, serum concentrations of mesothelin (12, 13), fibulin-3 (14), HMGB1 (15), osteopontin (16), hyaluronic Acid (17), and microRNAs (18, 19) were found unsuitable as early biomarkers of MPM. Blood DNA methylation profile may be diagnostic, but further prospective validation is needed (20, 21). These findings suggest that the identification of other noninvasive screening biomarkers is warranted.

Volatile organic compounds (VOCs) in exhaled breath (22, 23), known as breathomics, have been found to reflect pathophysiological processes, and measurements of VOCs are easy, noninvasive methods for detecting various diseases (24–26). Breath VOCs are products of cellular metabolism mainly associated with oxidative stress, including products of lipid peroxidation (27), inflammation, and cellular metabolism or degradation (28). Types and concentrations of endogenous VOCs have been found to differ between diseased patients and healthy controls (HCs) (26, 29). Methods used to analyze VOCs from various biological sources include gas chromatography (GC) coupled with mass spectrometry (MS), selected ion flow tube MS (30, 31), GC-ion mobility MS (32), GC/time-of-flight MS (33), and proton transfer reaction MS (34), with some of these methods identifying VOCs that can act as cancer biomarkers. In addition, the electronic nose (e-Nose) is an analytical technique that captures comprehensive information from all components of exhaled breath, rather than identifying specific biomarkers (35, 36).

Analytical and/or sensor techniques have been utilized to characterize MPM- related VOCs present in breath samples, with the results of these assays analyzed statistically using specific data mining methods. Although cyclohexane has been reported to differentiate MPM patients from HCs (37, 38), and several VOCs (such as P3, P5, P50, and P71) have shown high sensitivity and negative predictive value in differentiating MPM patients from AEx individuals (39, 40), the small sample sizes in these trials limited the generalizability of the results. The present study therefore conducted a meta-analysis of previous studies evaluating the association between VOCs detected in exhaled breath and a diagnosis of MPM.

## Materials and methods

### Inclusion and exclusion criteria

The present study was conducted under the PRISMA guidelines (41). Studies on exhaled VOCs for MPM diagnosis were included if they were (1) clinical studies; (2) involved adult patients diagnosed with MPM; (3) included the detection of exhaled VOCs in these subjects; (4) utilized pathological or cytological methods as the standard for MPM diagnosis; and (5) included either HCs or AEx controls and reported sensitivity, specificity, and true positive (Tp), false positive (Fp), true negative (Tn), and false negative (Fn) rates (determined in the initial studies or calculated from their data). Studies were excluded if they (1) did not provide specific experimental data; (2) were commentaries, reviews, letters, or meta articles; (3) reported changes in VOCs before and after MPM treatment; (4) were published in a language other than English or were unpublished research, or (5) involved patients with other tumor types or non-exhaled samples.

### Search strategies

Publicly available databases, including the Web of Science, EMBASE, PubMed, and the Cochrane Library were thoroughly searched for studies published up to August 30, 2024 without restrictions on region and language. Articles were retrieved using MeSH terms and free words related to “Malignant pleural mesothelioma” and “volatile organic compounds”. Details of the search strategy are displayed in **Supplementary File S1**. References in relevant reviews were also screened to obtain related information.

### Study selection

Studies retrieved from the search by two independent reviewers were imported into EndNote21 and duplicates were removed. The titles and abstracts of initially eligible studies were screened. Identified studies were further screened by reading their full texts. Any disagreements between reviewers were resolved by consultation with a third reviewer.

### Data extraction

Data were extracted by two reviewers separately, with any disagreements between reviewers resolved by consensus. The following data were recorded: first author’s family name, location, year of publication, study design, number of participants, mean age of participants, VOC analytical methods, sampling technique, sample volume, cancer stages, histology types, analytic methodology, outcomes (sensitivity, specificity, Tp), and identified VOCs (if provided).

### Quality assessment

Study quality was evaluated using four domains of the Quality Assessment of Diagnostic Accuracy Studies (QUADAS-2) checklist tool: patient selection, index test, reference standard, and flow and timing (42). Each component was appraised for risk of bias, with the first three domains also evaluated for their clinical applicability. If the answer to all key questions in a domain was ‘yes’, the risk of bias would be rated as low. If an answer was ‘no’, the risk of bias was judged to be high. Unclear answers were defined as unknown risk. Two reviewers assessed study quality independently, with disagreements resolved by consensus. The quality of included articles was evaluated using Cochrane’s RevMan 5.4 software.

### Statistical analysis

All diagnostic data were analyzed by Meta-Disc1.4 and Stata17.0 software using a MIDAS module of a bivariable mixed-effects model. The results of bivariate meta-analysis were summarized by Forest plots that contained confidence regions for sensitivity, specificity, positive likelihood ratio (PLR), negative likelihood ratio (NLR), diagnostic score (DS), diagnostic odds ratio (DOR), 95% confidence interval (CI), and summary receiver operating characteristics (SROCs) curves. Higher DS and DOR values were indicative of better diagnostic quality. Areas under the SROC curves (AUC) were calculated, with AUC values of 0.5–0.7, 0.7–0.9, and 0.9–1.0 indicating low, medium, and high diagnostic efficiency, respectively. The stability of the results was evaluated by sensitivity analysis, in which the effect of deletion of a single study on the combined results was evaluated. Because the threshold effect was a source of heterogeneity, Spearman correlation analysis was performed, with a negative coefficient indicating a threshold effect. Statistical heterogeneity due to non-threshold effects was tested by the Q test and I^2^ test, with I^2^ ≥ 50% indicating notable heterogeneity. If notable heterogeneity was observed, a random-effects model was utilized; otherwise, a fixed-effect model was utilized. If heterogeneity was high, its sources were determined by meta-regression and subgroup analyses. Publication bias was evaluated using Deeks funnel plot symmetry test, with *P* < 0.05 denoting statistical significance.

## Results

### Study search and selection

**Figure 1:**
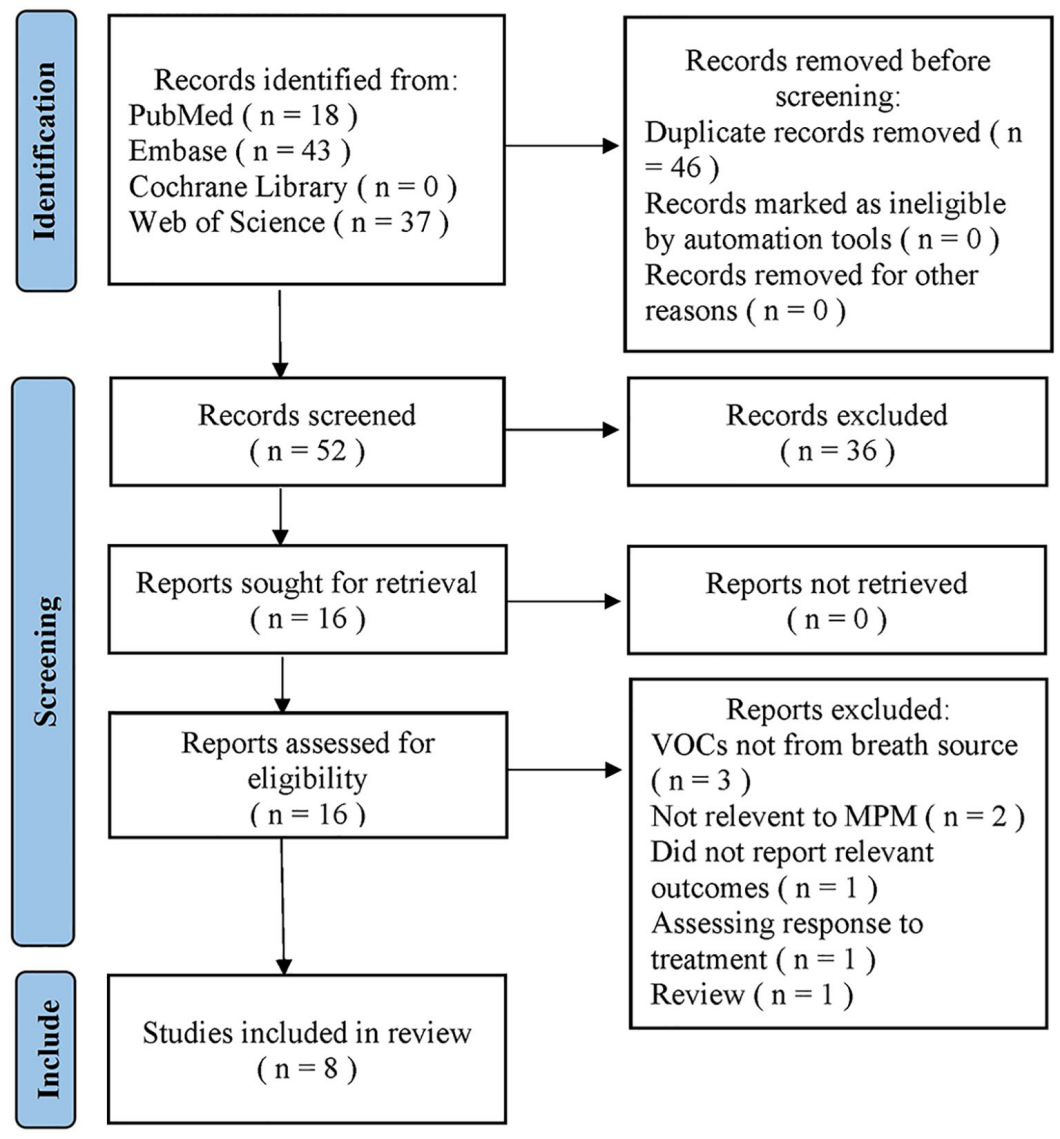
Flowchart for the selection of eligible studies.

A search of the four databases retrieved 98 articles; of these, 46 were duplicates and were removed by Endnote21, and 36 were deleted based on their titles and abstracts. After a full-text review, eight articles were excluded, leaving eight eligible articles for meta-analysis (38–40, 43–47). The study selection procedure is illustrated in **Figure 1**. The reports excluded are detailed in **Supplementary Files S3**.

### Characteristics of selected articles

The eight studies included a total of 859 subjects (38–40, 43–47). Five of these studies (38–40, 43, 44) had at least two sets of data, resulting in 24 sets of complete data from the eight included articles (38–40, 43–47). The number of MPM patients per study ranged from 6 to 52. Of the 859 subjects, 670 were non-MPM controls, including HCs, asymptomatic former asbestos workers, patients with benign asbestos-related diseases, patients with benign non-asbestos-related lung diseases, and patients with lung cancer. The basic traits of the study subjects are summarized in **Table 1**. Because of the small sample sizes in each study, most were subjected to cross-validation methods for verification. All eight articles were published between 2012 and 2023.

**Table 1:** Basic traits of the studies of VOCs in MPM detection.

Author(ref)	Year	Country	Cancer Stage	Histology	No. Patients (Cancer/all)	Age (years)	Analytical Method	Sampling Technique	Volume	Statistics	Sensitivity (%)	Specificity (%)	Target marker
Chapman et al (44)	2012	Australia	1, 19, X, X	NR	20 / 80	68.10 ± 12.02	CPA e-Nose (Cyranose 320)	One-way non- rebreathing valve, Bag	2-L gas impermeable bag	PCA	90	91	Profile (*vs*. HCs)
											90	88	Profile (*vs*. HCs *vs*. ARD)
Gilio et al. (45)	2020	Italy	NR	NR	14 / 39	62.05 ± 22.65	TD-GC-MS	Bag, TD	3 L-Tedlar bags	Nonparametric test	92.7	84.2	10 VOCs (*vs*. HCs)
Dragonieri et al. (43)	2012	Italy	8, 3, 2, X	9 epithelial, 2 biphasic, 2 desmoplastic	13 / 39	60.10 ± 14.11	e-Nose (Cyranose 320)	VOC filter, Bag	5-L Tedlar bag	Student’s t-test; PCA; CDA	92.3	69.2	Profile (*vs*. HCs)
											92.3	85.7	Profile (*vs*. AEx)
Janssens et al. (46)	2022	Belgium	NR	NR	47 / 123	66.90 ± 9.21	MCC/IMS	Disposable mouthpiece, VOC filter	10 mL (background)	Lasso Regression, LOOCV	91.5	61.8	11 VOCs (*vs*. AEx)
Lamote et al. (38)	2017	Belgium	NR	NR	14 / 64	58.00 ± 8.20	GC-MS	VOC filter, Bag, TD	10 L Tedlar bags	Lasso Regression; PCA; Shapiro-Wilk test; Ch²-test	100	91	19 VOCs (*vs*. AEx + ARD)
											92.9	100	17 VOCs (*vs*. AEx)
											78.6	80	7 VOCs (*vs*. ARD)
											64.3	78.6	8 VOCs (*vs*. HCs)
							e-Nose				81.5	54.5	Profile (*vs*. AEx + ARD)
											80	63.6	Profile (*vs*. AEx)
											75	63.6	Profile (*vs*. ARD)
											66.7	63.6	Profile (*vs*. HCs)
Lamote et al. (40)	2017	Belgium	NR	NR	52 / 330	59.80 ± 14.05	MCC/IMS	Disposable mouthpiece, Bag	10 mL	Lasso regression	88.5	42.3	16 VOCs (*vs*. HCs)
											86.5	89.8	13 VOCs (*vs*. AEx)
											88.5	73.2	19 VOCs (*vs*. ARD)
											94.2	80	19 VOCs (*vs*. AEx + ARD)
											71.2	87.1	9 VOCs (*vs*. BLD)
											73.1	71.4	32 VOCs (*vs*. LC)
Lamote et al. (39)	2016	Belgium	NR	NR	23 / 66	59.50 ± 10.72	MCC/IMS	Disposable mouthpiece, VOC filter	10 mL	Lasso regression	87	70	4 VOCs (*vs*. AEx + HCs)
											87	86	6 VOCs (*vs*. AEx)
											96	67	2 VOCs (*vs*. HCs)
Zwijsen et al. (47)	2023	Belgium	NR	NR	6 / 118	61.10 ± 7.05	MCC/IMS	Disposable mouthpiece, VOC filter	10 mL	Lasso regression	100	30	4 VOCs (*vs*. AEx)

AEx, asbestos-exposed; ARD, benign asbestos related diseases; BLD, benign non-asbestos related lung diseases; CAP, canonical principal coordinate analysis; CDA, canonical discriminant analysis; e-Nose, electronic nose; GC-MS, gas chromatography–mass spectrometry; HCs, healthy controls; LC, primary lung cancer; LOOCV, leave one-out cross-validation; MCC/IMS, multicapillary column/ion mobility spectrometry; NR, not reported; PCA, principal component analysis; TD, thermal desorption; TD-GC-MS, thermal desorption-gas chromatography–mass spectrometry; VOC, volatile organic compound.

Exhaled VOCs were analyzed using various methods. Exhaled samples, ranging in volume from 10 mL to 10 L, were harvested temporarily in Tedlar bags or cans and then analyzed directly. Samples in four studies were analyzed by MCC/IMS (39, 40, 46, 47) and samples in three studies were analyzed by e-Nose (Cyranose 320) (38, 43, 44). In one of the latter studies, samples were analyzed by GS coupled to MS and e-Nose (38). These analyses found that 149 VOCs were associated with a diagnosis of MPM, with most of these VOCs being aromatic compounds, alkanes, and alkenes (**Table 2**). The most highly detected compounds, identified in at least two studies, were cyclohexane, toluene, limonene, diethyl ether, xylene, acetophenone, 2-ethyl-1-hexanol, hexane, alpha-pinene, beta-pinene, P3, P5, P50, P54, and P84. These VOCs had a sensitivity of 71.2% to 100% and a specificity of 30% to 91% in the diagnosis of MPM.

**Table 2:** Significant VOCs identified in patients with MPM.

Study (ref)	Significant VOCs
Gilio et al., 2020 (45)	10 VOCs: acetophenone, 1-hexonol-2-ethyl, α-pinene, p-benzoquinone, 2,2,4,6,6- pentamethyl-heptane, 1-propanol, benzene, benzonitrile, ethylbenzene, toluene
Janssens et al., 2022 (46)	11 VOCs: P1, P7, P9, P15, P21, P26, P84, P88, P101, P122, P236
Lamote et al., 2017 (38)	41VOCs: VOC I_K_931, VOC I_K_720, VOC I_K_679, VOC I_K_1349, VOC I_K_1309, VOCI_K_ 1287, VOC I_K_1287, VOC I_K_1233, VOC I_K_1100, Tert-butylbenzene, Propylbenzene, Phenol, Nonane, Nonanal, n-Butylbenzene, Naphthalene, Methylcyclopentane, Methylbenzoate, m/p-xylene, Linalool, Limonene, Isothiocyanatocyclohexane, Isoprene, Hexane, Hexamethyldisiloxane, Furfural, Ethanol, Diethylether, Cyclohexane, Chloroform, Bromobenzene, Beta-pinene, Benzonitrile, 3- methylpentane,2-methyl-1-propanol, 2-ethyl-1-hexanol, 2,2,4-trimethylpentane, 1,2- dichlorobenzene, 1,3-dichlorobenzene, 1,2,4-trichlorobenzene, 1,2,3-trichlorobenzene
Lamote et al., 2017 (40)	93 VOCs: P99, P94, P92, P9, P88, P84, P83, P8, P78, P73, P70, P7, P65, P48, P43, P42, P4, P37, P34, P3, P28, P26, P248, P245, P244, P243, P240, P237, P236, P235, P231, P225, P224, P223, P222, P221, P220, P218, P216, P215, P212, P21, P208, P207, P203, P195, P187, P186, P185, P181, P178, P177, P176, P173, P167, P164, P161, P159, P156, P153, P151, P150, P15, P145, P142, P137, P136, P132, P130, P129, P127, P126, P123, P122, P121, P120, P119, P118, P117, P116, P115, P114, P112, P110, P108, P107, P104, P103, P102, P101, P10, P1, P0
Lamote et al., 2016 (39)	7 VOCs: P84, P71, P54, P50, P5, P30, P3

In recent reports, these codes and abbreviations were routinely used based on different detection methods.

### Quality assessment

**Figure 2:**
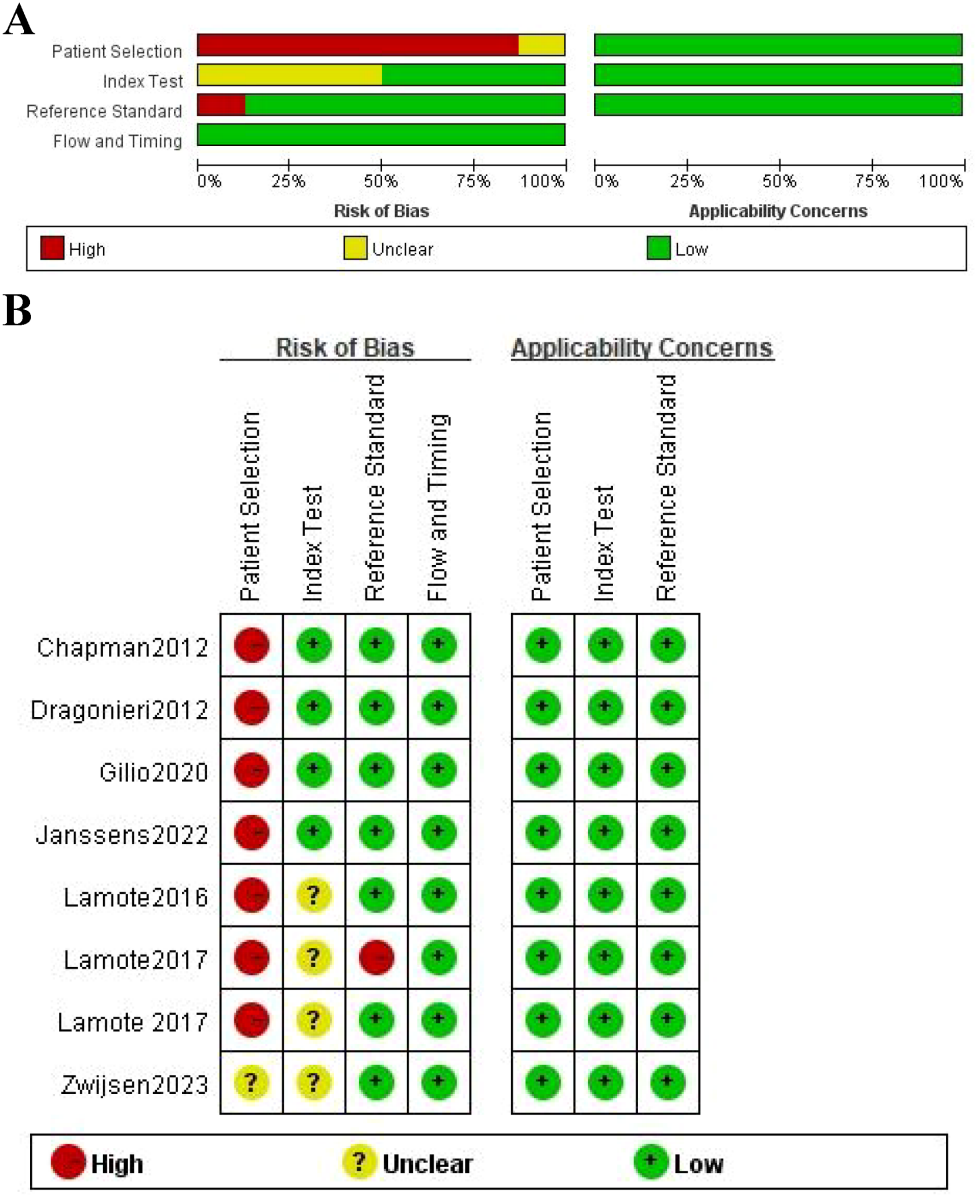
Quality assessment using the QUADAS-2 tool. **(A)** risk of bias graph; **(B)** risk of bias summary.

The quality of the eight included studies was assessed by QUADAS-2 (**Figure 2**). Seven (87.5%) of these studies had a case-control design, which may have introduced selection bias as this design typically includes only selected cases and controls, potentially not representing the broader patient population. Such a design could affect the external validity and generalizability of the results. Therefore, these studies were categorized as having a high risk of bias (red). One study (12.5%) did not provide sufficient information and was thus rated as having an unclear risk (yellow). On the index test, four studies (50%) had no significant applicability concern, whereas the other four (50%) had an unknown concern. That means the suitability of the test for the studied population was uncertain, which could influence the accuracy of the test’s performance. These studies were thus rated with an unclear risk of bias (yellow). Reference standard results in seven studies (87.5%) were interpreted without knowing the index test results, which minimizes the risk of bias in the evaluation process. However, one study (12.5%) were not blinded, increasing the potential for bias. These studies were classified based on their respective biases: high risk for the unblinded study (red) and low risk for the others (green). There were no pronounced applicability concerns for flow and timing. All studies had a clear follow-up period and no major timing-related biases, so these studies were marked as green (low risk). The overall quality of all studies in patient selection, index test, and reference standards was moderately high.

### MPM and health control

**Figure 3:**
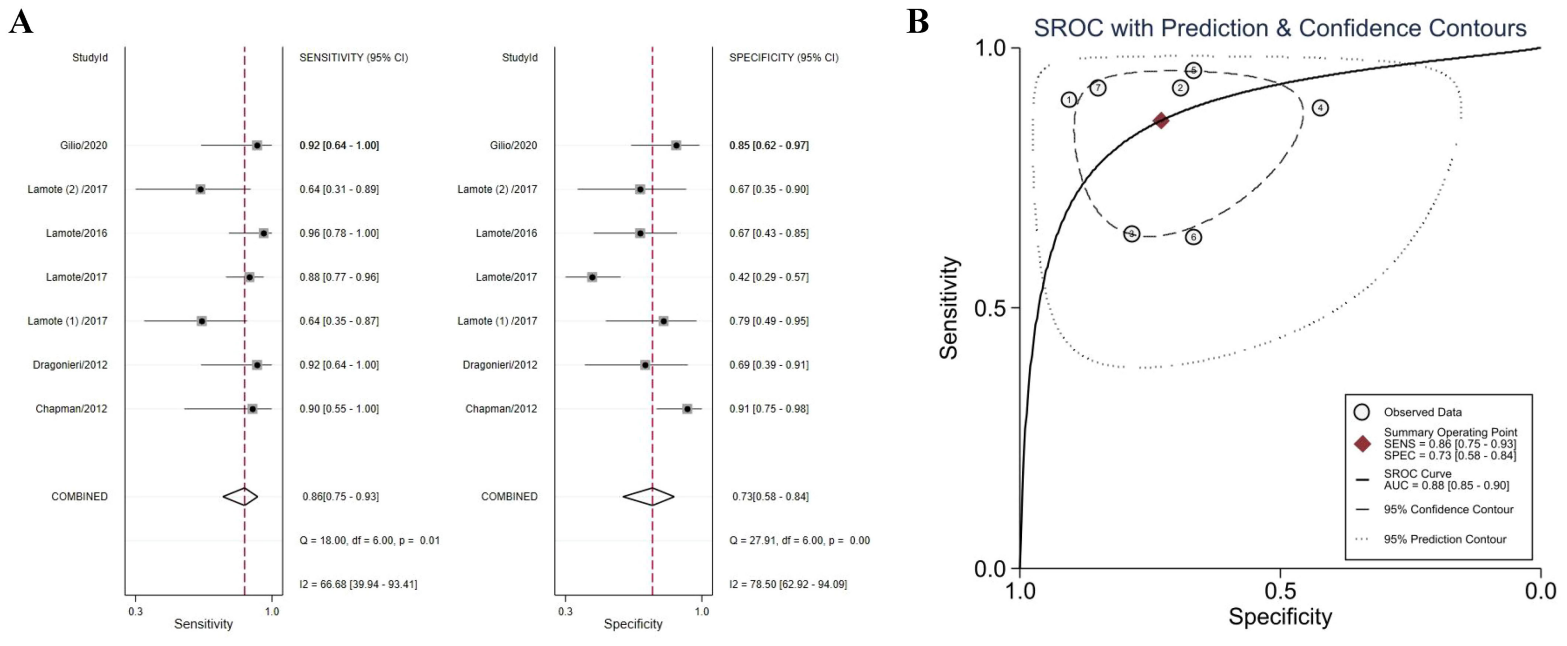
Forest plot **(A)** and SROC curve **(B)** of volatile organic compounds for the detection of MPM (*vs.* HCs). Abbreviations: HCs, healthy controls; SROC, summary receiver operating characteristic.

Six studies directly compared MPM patients with HCs to identify VOCs that could distinguish between the two (38–40, 43–45). The meta-analysis found that VOCs could distinguish between MPM patients and HCs with a sensitivity of 86% (95% CI 75–93%), a specificity of 73% (95% CI 58–84%) (**Figure 3A**), and an AUC of 0.88 (95% CI 85–90%) (**Figure 3B**). Both specificity (I^2^ = 78.50%) and sensitivity (I^2^ = 66.68%) were heterogeneous. Accurate estimated points were not distributed in a “shoulder arm” pattern, suggesting no threshold effect, consistent with the results of Spearman correlation analysis (*P* = 0.76).

### MPM and AEx

**Figure 4:**
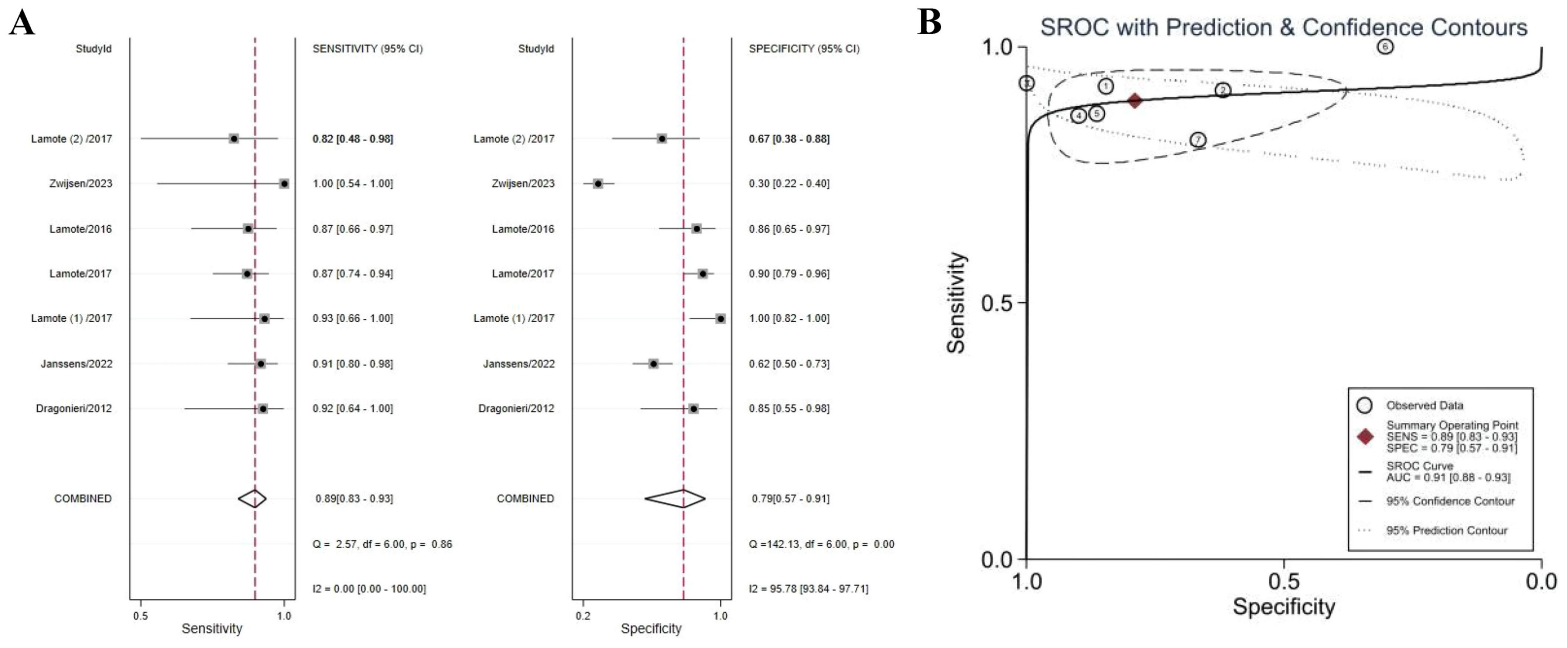
Forest plot **(A)** and SROC curve **(B)** of volatile organic compounds for the detection of MPM (*vs.* AEx). Abbreviations: AEx, asbestos-exposed; SROC, summary receiver operating characteristic.

Six studies compared VOCs of MPM patients with AEx subjects (38–40, 43, 46, 47). The meta-analysis found MPM patients could be distinguished from subjects with AEx with a sensitivity of 0.89 (95% CI 0.83–0.93), a specificity of 0.79 (95% CI 0.57–0.91), and an AUC of 0.91 (95% CI 0.88–0.93), indicating outstanding diagnostic performance (**Figures 4A, B**). Specificity (I^2^ = 95.78%) was highly heterogeneous. Accurate estimated points were distributed in a “shoulder arm” pattern, but Spearman correlation analysis (*P* = 0.29) found that heterogeneity was not caused by a threshold effect.

### Subgroup analysis

Subgroup analysis by location (Europe) showed notable heterogeneity in specificity (*P* = 0.05) (**Supplementary Files S2**). VOCs were found to distinguish MPM patients from HCs in Europe with a sensitivity of 0.85 (95% CI 0.76–0.94) and a specificity of 0.67 (95% CI 0.54–0.81). The e-Nose, however, was not a major source of heterogeneity.

### Sensitivity analysis and publication bias

**Figure 5:**
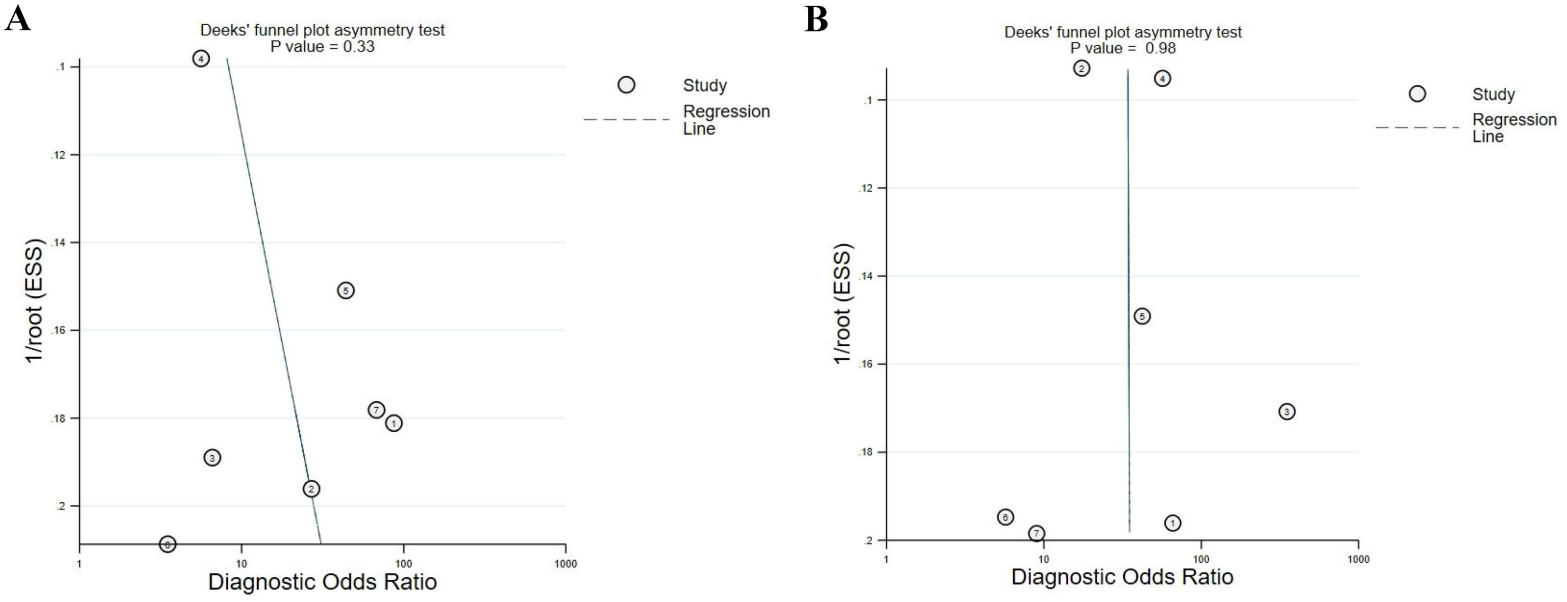
Deeks funnel plot of included articles, showing a lack of publication bias. **(A)** MPM vs. HCs ; **(B)** MPM vs. AEx.

A sensitivity analysis suggested the impact of each article on the combined results was acceptable and the overall results were robust. Deeks funnel plots showed no marked published biases (MPM *vs* HCs, *P* = 0.33; MPM *vs* AEx, *P* = 0.98), although these findings were limited by the small number of studies included in this analysis (**Figure 5**).

## Discussion

The present meta-analysis, consisting of eight studies with 189 MPM patients and 670 control subjects, showed that exhaled VOCs could distinguish MPM patients from HCs and AEx subjects with high accuracy. Exhaled VOCs were found to distinguish MPM patients from HCs with a sensitivity of 86% (95% CI 75–93%), a specificity of 73% (95% CI 58–84%), and an AUC of 0.88 (95% CI 85–90%). In addition, VOCs could distinguish MPM patients from AEx subjects with a sensitivity of 0.89 (95% CI 0.83–0.93), a specificity of 0.79 (95% CI 0.57–0.91), and an AUC of 0.91 (95% CI 0.88–0.93). Subgroup analysis by location showed that VOCs had a specificity of 0.67 in Europe and a specificity of 0.9 in non-European locations.

These findings suggest that exhaled VOCs may have promise in the diagnosis of MPM. The relevant mechanism is considered to be ‘oxidative stress’, which has been well described as the underlying mechanism for the pathogenesis of many cancers (48, 49). Furthermore, Inflammatory cells promote tumor development and a change in cell metabolism is to be expected (50). Since asbestos fibers induce chronic inflammation and oxidative stress that leads to MPM, this will ultimately lead to a change in the VOC production and proposes that VOCs can be used as noninvasive diagnostic biomarkers for disease. Because cancer-related VOCs are released from tissues into the bloodstream and ultimately exhaled through alveolar gas exchange, analysis of the composition and concentration of exhaled VOCs may serve as an accessible, non-invasive, low-cost method of evaluating metabolic and pathologic changes in cancer patients (26, 51). VOC profiles have been analyzed to establish distinctive fingerprint/odor signatures linked to individual diseases, potentially aiding in early diagnosis and enhancing survival.

Several diagnostic tools have demonstrated promising outcomes in differentiating between patients and HCs. In assessing the ability of VOCs to distinguish HCs from MPM patients, MCC-IMS exhibited the lowest accuracy (65%) (38), e-Nose exhibited the highest accuracy (95%) (44), and GC-MS exhibited intermediate accuracy (71%) (40). Differences were also observed in assessments of the ability of VOCs to differentiate between MPM patients and subjects with AEx, with MCC-IMS having an accuracy of 73% to 88% (39, 40, 46, 47), GC-MS having an accuracy of 97%, and e-Nose having an accuracy of 73-81% (38, 43). Because the risk of MPM is highest in individuals with asbestos-related diseases (ARD), there is interest in employing breath tests as a screening tool. When MPM patients were compared with a combined group of subjects with AEx and ARD, e-Nose yielded the lowest accuracy (74%), GC-MS yielded the highest accuracy (94%) (38), and MCC-IMS had intermediate accuracy (85%) (40). These findings may be due to the ability of the e-Nose to detect VOC patterns rather than identifying specific VOCs, whereas GC-MS and MCC-IMS can identify individual VOCs.

MPM, which is frequently associated with AEx, is characterized by local inflammatory conditions that result in the generation of cytokines and reactive oxygen species (ROS) (52, 53). The exhaled breath of MPM patients contains high concentrations of oxidized organic compounds, including acetophenone, p-benzoquinone, propanol, 1-hexonol-2-ethyl, cyclohexane, benzaldehyde, trimethyl benzene, and limonene (37, 40, 43, 45). Oxidized compounds suggest a cytochrome p450 polymorphism (54), whereas methylated compounds have been linked to methylation processes involved in tumorigenesis (55). Cyclohexane alone has been reported to significantly distinguish MPM patients and HCs (37), suggesting a link between the degradation of xenobiotic agents and neoplastic processes (56, 57). Nevertheless, the precise mechanisms underlying the endogenous origin of these organic compounds remain unclear. Acetophenone may be connected to a deficiency in the enzyme phenylalanine hydroxylase through oxidative stress, producing phenyl ketones via an alternative pathway and ultimately influencing the catalytic conversion of L-Phe to L-Tyr (58). The alcohol 1-hexonol-2-ethyl likely arises from alkane metabolism (59). Alkanes are generated through lipid peroxidation, a consequence of oxidative stress, suggesting that the increased concentration of this alcohol may stem from enhanced oxidative stress and CYP450 (60). Hydrocarbon compounds such as ethylbenzene, benzene, and toluene are exogenous pollutants associated with tobacco smoke, environmental pollution, and radiation exposure. Many cancer patients have a history of heavy smoking and/or sustained occupational exposure to exogenous pollutants, which can accumulate in fatty tissues. These absorbed compounds can cause peroxidative damage to polyunsaturated fatty acids, proteins, and DNA, facilitating the development of age-dependent diseases including cancer.

Sources of heterogeneity can include threshold and non-threshold effects. The present meta-analysis found no heterogeneity due to threshold effects. Subgroup analysis and meta-regression found that heterogeneity may have been due to subject location (Europe), possibly due to a lower number of patients in non-European study, which may affect the stability and reliability of specificity estimates. Moreover, the lack of grouping of VOCs into those arising endogenously and from background environmental contamination, but this was likely to improve accuracy (44). These methodological variations, combined with differences in regional MPM incidence rates, could partially explain why European studies tended to report higher specificity compared to studies from other regions, such as Australia. While methodological differences in detection techniques may also play a role, the combination of these factors suggests that standardizing protocols and increasing sample sizes in future studies could help mitigate these discrepancies and improve diagnostic accuracy. Interestingly, the analytical techniques used to detect VOCs had no effect on outcomes. GC coupled with MS is considered the standard method, as it allows for quantitative and qualitative analyses (61). This method, however, is time-consuming and requires extensive experience. The e-Nose is an analytical technique that offers a complete analysis of all exhaled breath components, rather than identifying specific biomarkers (35, 36). This approach results in lower discriminative power due to its reduced specificity. However, sensor arrays are more affordable, portable, and capable of delivering real-time results, making them more suitable for point-of-care applications than GC-MS. Nonetheless, the accuracy of the e-Nose is substantially influenced by both endogenous and exogenous factors, highlighting the necessity for further investigation (62, 63). Another method for breath analysis is MCC/IMS, although this method cannot precisely identify specific VOCs and offers only a pseudo identification (64). It brings together the strengths of both GC-MS and e-Nose, offering rapid, affordable, and portable analysis with high sensitivity, while efficiently identifying volatile compounds in complex samples. Most of the studies included in this meta-analysis, however, analyzed VOCs using MCC/IMS techniques, with sample sizes in the included studies differing markedly.

This study had several limitations, including the limited sample size, which may have reduced its statistical power. Few studies to date have analyzed the connections among MPM stages, subtypes, related lung diseases, and exhaled VOCs, and the scarcity of these data will affect statistical analyses. Five of the included studies were performed by the same research team, which may have introduced selection bias. Interfering factors in exhaled breath samples, such as the subjects’ diet, medication, exercise, sample collection environment, sample containers, and the cleanliness and contamination of real-time monitoring equipment, also varied across the studies included in our analysis. Moreover, most of the included studies were retrospective in design. Prospective longitudinal trials of cancer-specific biomarkers (e.g., VOCs) in MPM patients are required to accurately assess the correlation between VOCs and disease severity. To improve specificity, it is essential to explore endogenous VOCs in MPM and correlate them with mesothelin (65, 66). By comparing VOCs in exhaled breath with those in the headspace gases and pleural fluids of mesothelioma cell lines, VOCs can be linked to the pathophysiology of MPM (38). Finally, all included studies were published in English, which may have resulted in additional selection bias.

## Conclusions

MPM is a malignant tumor with a high mortality rate, with prompt detection and treatment required to enhance survival rates. Exhaled VOCs offer a non-invasive method for MPM diagnosis and address current screening limitations and guide further diagnostic advancements. To our knowledge, the present meta-analysis is the first to quantitatively evaluate VOCs as a promising novel biomarker for MPM diagnosis. Preliminary findings indicate that exhaled breath VOCs can effectively differentiate between MPM patients and HCs, as well as between MPM patients and AEx individuals. However, substantial heterogeneity across studies, including variability in VOC detection techniques and sample sizes, highlights the need for further investigation. Future studies should include larger, multi-center cohorts with standardized protocols to validate these findings. Additionally, a deeper exploration into the molecular mechanisms underlying VOC production in MPM could lead to more specific biomarkers, improving diagnostic accuracy and clinical applicability.

## Data Availability

The original contributions presented in the study are included in the article/**Supplementary Material**. Further inquiries can be directed to the corresponding author.
