# Supplementary material for: Volatile organic compounds in exhaled human breath for the diagnosis of malignant pleural mesothelioma: a meta-analysis
Source: Front Oncol. 2025 May 28;15:1537767. doi: 10.3389/fonc.2025.1537767 (PMC12151828; doi:10.3389/fonc.2025.1537767)
Supplement: Supplementary file 1 [file DataSheet1.docx]

**Supplemental Files S1.** Retrieval strategy

**PubMed: 18 records**

#1: Mesothelioma, Malignant[MeSH Terms]

#2:(((((((Mesothelioma, Malignant[Title/Abstract]) OR (Malignant Mesothelioma*[Title/Abstract])) OR (Malignant Pleural Mesothelioma*[Title/Abstract])) OR (mesothelioma of pleura[Title/Abstract])) OR (mesothelioma of the pleura[Title/Abstract])) OR (mesothelioma pleurae[Title/Abstract])) OR (pleural mesothelioma[Title/Abstract])) OR (pleura mesothelioma[Title/Abstract])

#3:(Mesothelioma, Malignant[MeSH Terms]) OR ((((((((Mesothelioma, Malignant[Title/Abstract]) OR (Malignant Mesothelioma*[Title/Abstract])) OR (Malignant Pleural Mesothelioma*[Title/Abstract])) OR (mesothelioma of pleura[Title/Abstract])) OR (mesothelioma of the pleura[Title/Abstract])) OR (mesothelioma pleurae[Title/Abstract])) OR (pleural mesothelioma[Title/Abstract])) OR (pleura mesothelioma[Title/Abstract]))

#4:Volatile Organic Compounds[MeSH Terms]

#5:(Volatile Organic Compounds[Title/Abstract]) OR (Volatile Organic Compound*[Title/Abstract])

#6: (Volatile Organic Compounds[MeSH Terms]) OR ((Volatile Organic Compounds[Title/Abstract]) OR (Volatile Organic Compound*[Title/Abstract]))

#7: ((Mesothelioma, Malignant[MeSH Terms]) OR ((((((((Mesothelioma, Malignant[Title/Abstract]) OR (Malignant Mesothelioma*[Title/Abstract])) OR (Malignant Pleural Mesothelioma*[Title/Abstract])) OR (mesothelioma of pleura[Title/Abstract])) OR (mesothelioma of the pleura[Title/Abstract])) OR (mesothelioma pleurae[Title/Abstract])) OR (pleural mesothelioma[Title/Abstract])) OR (pleura mesothelioma[Title/Abstract]))) AND ((Volatile Organic Compounds[MeSH Terms]) OR ((Volatile Organic Compounds[Title/Abstract]) OR (Volatile Organic Compound*[Title/Abstract])))

**Embase: 43 records**

#1：pleura mesothelioma'/exp

#2：'pleura mesothelioma'/exp OR 'pleura mesothelioma' OR (('pleura'/exp OR pleura) AND ('mesothelioma'/exp OR mesothelioma)) OR 'malignant mesothelioma*':ab,ti OR 'malignant pleural mesothelioma*':ab,ti OR 'mesothelioma of pleura':ab,ti OR 'mesothelioma of the pleura':ab,ti OR 'mesothelioma pleurae':ab,ti OR 'pleural mesothelioma':ab,ti OR 'pleura mesothelioma':ab,ti

#3：#1 OR #2

#4: 'volatile organic compound'/exp

#5:'volatile organic compound'/exp OR 'volatile organic compound' OR (volatile AND organic AND ('compound'/exp OR compound)) OR 'volatile organic compound*':ab,ti

#6:#4 OR #5

#7:#3 AND #6

**Cochrane Library:0 records**

#1 MeSH descriptor: [Mesothelioma, Malignant] explode all trees 151

#2 (Mesothelioma, Malignant):ti,ab,kw OR (Malignant Mesothelioma*):ti,ab,kw OR (Malignant Pleural Mesothelioma*):ti,ab,kw OR (mesothelioma of pleura):ti,ab,kw OR (mesothelioma of the pleura):ti,ab,kw

#3 (mesothelioma pleurae):ti,ab,kw OR (pleural mesothelioma):ti,ab,kw OR (pleura mesothelioma):ti,ab,kw

#4 #1 OR #2 OR #3

#5 MeSH descriptor: [Volatile Organic Compounds] explode all trees

#6 (Volatile Organic Compounds):ti,ab,kw OR (Volatile Organic Compound*):ti,ab,kw

#7 #5 OR #6

#8 #4 AND #7

**Web of Science: 37 records**

TS="Mesothelioma, Malignant (Topic) OR Malignant Mesothelioma* (Topic) OR Malignant Pleural Mesothelioma* (Topic) OR mesothelioma of pleura (Topic) OR mesothelioma of the pleura (Topic) OR mesothelioma pleurae (Topic) OR pleural mesothelioma (Topic) OR pleura mesothelioma (Topic) "

TS="Volatile Organic Compounds (Topic) OR Volatile Organic Compound* (Topic) "

TS="#4 AND #2 "
