# Supplementary figures and images for: Volatile organic compounds in exhaled human breath for the diagnosis of malignant pleural mesothelioma: a meta-analysis
Source: Front Oncol. 2025 May 28;15:1537767. doi: 10.3389/fonc.2025.1537767 (PMC12151828; doi:10.3389/fonc.2025.1537767)

**Supplemental Files S2.** Subgroup analysis by location (Europe vs non-Europe).


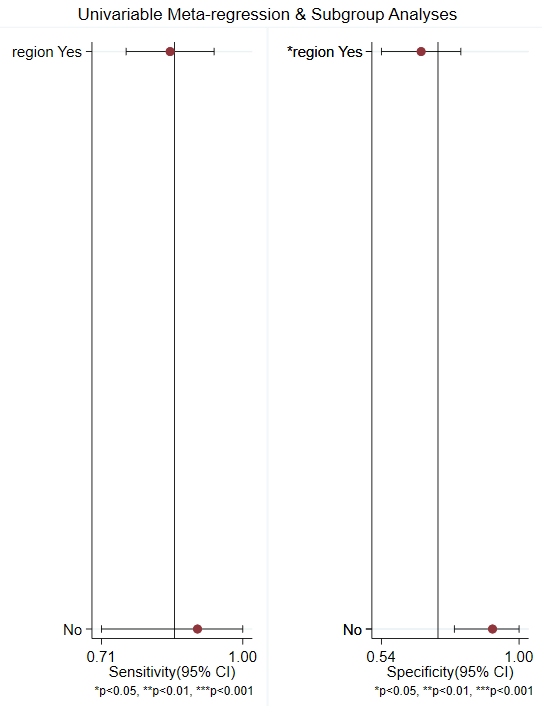

Supplement: Supplementary file 2 [file DataSheet2.docx]
