# Supplementary material for: Volatile organic compounds in exhaled human breath for the diagnosis of malignant pleural mesothelioma: a meta-analysis
Source: Front Oncol. 2025 May 28;15:1537767. doi: 10.3389/fonc.2025.1537767 (PMC12151828; doi:10.3389/fonc.2025.1537767)
Supplement: Supplementary file 3 [file Table1.docx]

**Supplemental Files S3.** Exclusion list.

| Number | Title |  | Author | Exclusion criteria |
| --- | --- | --- | --- | --- |
| 1 | Headspace Volatile Organic Compound Profiling of Pleural Mesothelioma and Lung Cancer Cell Lines as Translational Bridge for Breath Research |  | E. Janssens | VOCs not from breath source |
| 2 | Volatile Organic Compound Analysis of Malignant Pleural Mesothelioma Chorioallantoic Membrane Xenografts |  | L. D. Little | VOCs not from breath source |
| 3 | Headspace analysis of mesothelioma cell lines differentiates biphasic and epithelioid sub-types |  | L. D. Little | VOCs not from breath source |
| 4 | Spray layer-by-layer assembly of POSS functionalized CNT quantum chemo-resistive sensors with tuneable selectivity and ppm resolution to VOC biomarkers |  | S. Nag | Not relevent to MPM |
| 5 | Increased Number of Volatile Organic Compounds Over Malignant Glottic Lesions |  | H. Shoffel-Havakuk | Not relevent to MPM |
| 6 | Chemical characterization of exhaled breath to differentiate between patients with malignant plueral mesothelioma from subjects with similar professional asbestos exposure |  | G. de Gennaro | Did not report relevant outcomes |
| 7 | Nose in malignant mesothelioma-Prediction of response to immune checkpoint inhibitor treatment |  | M. J. Disselhorst | Assessing response to treatment |
| 8 | Strengths, weaknesses, and opportunities of diagnostic breathomics in pleural mesothelioma-a hypothesis |  | K. Lamote, | Review |
